# Alkindi Sprinkle for Pediatric Patients With Primary Adrenocortical Insufficiency: A Narrative Review

**DOI:** 10.7759/cureus.56031

**Published:** 2024-03-12

**Authors:** Alan D Kaye, Munira E Khaled, Kristin Nicole Bembenick, John Lacey, Anamika Tandon, Rucha A Kelkar, Alyssa G Derouen, Corrado Ballaera, Debbie Chandler, Shahab Ahmadzadeh, Sahar Shekoohi, Giustino Varrassi

**Affiliations:** 1 Anesthesiology, Louisiana State University Health Sciences Center, Shreveport, USA; 2 School of Medicine, Louisiana State University Health Sciences Center, Shreveport, USA; 3 School of Medicine, Louisiana State University Health Sciences Center at New Orleans, New Orleans, USA; 4 School of Medicine, Medical University of South Carolina, Charleston, USA; 5 Pain Medicine, Paolo Procacci Foundation, Rome, ITA

**Keywords:** pediatric endocrinology, glucocorticoid replacement, mineralocorticoid deficiency, adrenal crisis, pediatric adrenal insufficiency, adrenocortical insufficiency, alkindi sprinkle

## Abstract

Adrenocortical insufficiency, also known as adrenal insufficiency (AI), is an endocrine disorder characterized by inadequate production of adrenal hormones, including glucocorticoids and mineralocorticoids (MCs). The condition can be categorized as primary, secondary, or tertiary AI, depending on the location of the defect. Classical symptoms of AI include weakness, fatigue, abdominal pain, tachycardia, hypotension, electrolyte imbalances, and hyperpigmentation. In children, the most common cause of AI is classical congenital adrenal hyperplasia, which results from a deficiency in the 21-hydroxylase enzyme. The 21-hydroxylase enzyme produces all steroids, such as cortisol and aldosterone. AI management primarily involves hormone replacement therapy, typically with oral hydrocortisone and MC supplementation. However, the administration of hydrocortisone to pediatric patients presents challenges related to the lack of available dose-appropriate formulations. Historically, crushed or split adult tablets were used for the pediatric treatment of AI, although this poses an increased risk of under- or overtreatment. Inadequate dosing in the pediatric population can adversely affect growth, development, and metabolic health. Alkindi Sprinkle is a pediatric-specific hydrocortisone oral granule preparation that manages cortisol levels to help facilitate accurate therapeutic dosing. Alkindi offers several advantages, including accurate dosing, taste masking, and ease of administration. The present investigation describes AI, the management of AI, and the treatment of pediatric AI using Alkindi Sprinkle, including clinical efficacy.

## Introduction and background

Adrenocortical insufficiency, also known as adrenal insufficiency (AI), is an endocrinological disorder characterized by inadequate production of adrenal hormones, such as glucocorticoids (GC) and mineralocorticoids (MC) [1]. AI can be classified as either primary, secondary (defect at the level of the pituitary gland), or tertiary (defect at the level of the hypothalamus) [1]. Primary AI (PAI) is most commonly related to autoimmune destruction of the adrenal gland or an inborn error of steroidogenesis. Secondary AI is rare and typically results from pituitary adenomas that suppress the adrenal gland from producing GC related to a negative feedback loop [2]. Tertiary AI is caused by exogenous steroid treatment.

Manifestations of AI are typically associated with salt-wasting that presents as generalized weakness, fatigue, abdominal pain, tachycardia, hypotension, hyponatremia, hyperkalemia, hypoglycemia, and hyperpigmentation (seen only in PAI). The most common cause of AI in children is classical congenital adrenal hyperplasia (CAH) [3]. Classical CAH is an autosomal recessive disease related to a deficiency in the 21-hydroxylase enzyme responsible for the production of all steroids. Overall, a decrease in 21-hydroxylase decreases cortisol, aldosterone, and androgen production [4].

This enzyme deficiency leads to a shift in steroid pathogenesis toward androgen production [4]. Regardless of classification, AI can be a serious, life-threatening condition if not properly managed. The goal of AI treatment is hormone replacement, primarily with oral hydrocortisone, to reduce hyperplasia and overproduction of androgens and MCs, and oral replacement of MCs [4].

While oral hydrocortisone has proven to be a reliable and efficient treatment for AI in adults, the pediatric population faces significant challenges with regard to administration and effectiveness. Administering hydrocortisone to children presents a unique challenge related to the differences in dosage requirements compared to adults. Hydrocortisone tablets are only available in 5, 10, or 20 mg; however, infants and children may require much smaller doses. In this regard, pediatric dosing has required the crushing of large adult tablets [5]. This practice introduces the risk of either overtreating or undertreating young patients, as obtaining the ideal hydrocortisone dosage becomes difficult to measure.

Inadequate dosing increases the risk of adverse side effects on growth, pubertal development, blood pressure, body mass index, cardiovascular disease, and bone density [6]. In a 2017 study, a majority of pediatric physicians rely on compounded adult medication because of a lack of licensed, dose-appropriate formulations for children with AI [7]. Recent advancements in AI treatments led to the development of Alkindi Sprinkle (development name: Infacort). Alkindi Sprinkles are hydrocortisone granules used as hormone replacement in AI, specifically targeted toward children with CAH [8].

Alkindi offers several potential advantages for the treatment of AI in children, such as accurate dosing, taste masking, ease of administration, and reducing the likelihood of an adrenal crisis. Alkindi is administered by opening the capsule filled with the granules and emptying onto soft foods (not mixed) or directly onto a child’s tongue and swallowed to be absorbed enterically to replace deficient hormone (cortisol) in the body [9,10]. The granule formulation, filled by a pharmaceutical manufacturer to an exact quality- and quantity-assured dose, ensures that pediatric patients with AI are being adequately treated [11]. Several clinical studies have evaluated Alkindi for the treatment of pediatric AI. These studies demonstrated the ability to provide adequate GC and MC replacement with a decreased risk of inadequate dosing. Overall, Alkindi has reduced the likelihood of an adrenal crisis and improved patient outcomes [7]. The present review, therefore, investigates physiological considerations in AI, management of the condition, pharmacological aspects of the drug Alkindi Sprinkle, and the treatment of AI using Alkindi Sprinkle, including clinical efficacy.

## Review

Adrenocortical insufficiency

Clinical Manifestations

AI represents a constellation of clinical manifestations that arise from the failure of the adrenal glands to function properly. AI is primarily defined by the failure of the adrenal glands to secrete GC; however, additional loss of MC and androgens is also seen in some cases of AI. The clinical symptoms seen in AI are based upon the degree of adrenal gland dysfunction, related to whether additional MCs and androgens are lost [12]. Depending on the etiology of adrenal malfunction, AI, specifically classical adrenal hyperplasia, can present acutely or gradually and is generally associated with hypotension, acute abdominal symptoms, and laboratory abnormalities [13]. The major finding seen in AI is an adrenal crisis, which is defined as a systolic blood pressure of less than 110 mmHg, syncope, shock, and volume depletion [12]. Other symptoms that can be seen that are less specific for AI are abdominal pain, fever, nausea, vomiting, abdominal rigidity, diarrhea, confusion, lethargy, and disorientation [12]. Laboratory values show significant electrolyte abnormalities such as hyponatremia, hyperkalemia, metabolic acidosis, azotemia, and anemia [14]. If the cause of adrenal dysfunction is a primary adrenal gland dysfunction rather than a central dysfunction, skin and mucosal hyperpigmentation will also be seen [12]. The most deleterious complication feared from AI is cardiovascular collapse due to loss of GCs and subsequent hypotension and shock [15].

Pathogenesis

Various etiologies can lead to the loss of adrenal gland hormone secretion. The most common pathogenesis is the destruction of all three layers of the adrenal cortex. This destruction is gradual and is first seen when the cortisol response to stress is suboptimal, precipitating an adrenal crisis [16]. This causes further destruction of the adrenal cortex, resulting in almost 100% loss of adrenal secretions. The most common cause of adrenal tissue destruction is autoimmune. In 90% of patients who presented with AI, antibodies against the 21-hydroxylase enzyme were found [16]. This autoimmune presentation is most seen sporadically and in isolation and is not associated with any other autoimmune disorders. It can also be linked to another autoimmune disease as part of autoimmune polyglandular syndromes (APS) [12]. APS type 1 is comprised of PAI, chronic mucocutaneous candidiasis, hypoparathyroidism, dental hypoplasia, pernicious anemia, alopecia, and primary gonadal failure [17]. APS type 2 is comprised of PAI, autoimmune thyroid disease, primary gonadal failure, type 1 diabetes, celiac disease, pernicious anemia, myasthenia gravis, and vitiligo [12]. Other causes of AI are external insults to the system and include adrenal hemorrhage, cancer, infections, and various drugs that can inhibit critical enzymes of the steroidogenesis pathway [16].

Diagnosis

In the setting of an acute adrenal crisis, treatment should not be delayed while waiting for confirmatory testing. For a more gradual onset, diagnostic blood samples of cortisol and adrenocorticotropic hormone (ACTH) should reveal whether the AI is ACTH dependent or independent. A low morning cortisol concentration (<100 nmol/L) along with a cortisol stimulation test can help confirm the diagnosis [18]. The gold standard of AI diagnosis in adults is the synacthen test, a cortisol stimulation test where 250 ug of ACTH is administered intramuscularly (IM) or intravenously (IV), followed by serum cortisol 30-60 minutes later, which is considered the gold standard in the detection of PAI. The synacthen test has a sensitivity of 92% [18,19]. In the setting of ACTH-dependent AI, a cortisol stimulation test will be negative, and low levels of both cortisol and ACTH will be seen. In the pediatric population, the most definitive test is morning serum cortisol levels. A morning cortisol level of <3 ug/dL indicates AI, whereas a morning cortisol level >14.5 ug/dL indicates a normal hypothalamic-pituitary-adrenal axis [12]. A PAI diagnosis is confirmed by a morning serum cortisol level of <18 mcg/dL in the presence of elevated ACTH and plasma renin activity [12].

Management of adrenocortical insufficiency

Treatment for adrenocortical insufficiency revolves around replacing the deficient GCs while avoiding overreplacement [18]. Short-acting oral hydrocortisone, typically at 15-25 mg daily, has been the cornerstone of replacement [18]. Typical dosing regimens should be tailored for body size and weight, as the total daily hydrocortisone requirement is dependent on body surface area [18]. Dosing is done throughout the day, with the first dose being taken immediately upon awakening and then two smaller doses being taken four to six hours apart. A dose of prednisolone 3-5 mg, taken once or split twice daily, can be used for longer-acting steroid replacement [18]. However, long-acting GC replacement regimens have been shown to have more adverse effects [20]. Medication such as dexamethasone, beclomethasone, and deflazacort should be avoided since their half-lives are long and they may result in overtreatment [18]. CAH requires a higher dose of GC replacement than PAI [20]. This is due to the fact that higher doses are needed in Addison’s disease to adequately suppress adrenal androgen production. The treatment of PAI also requires the replacement of MCs with fludrocortisone at a dose of 0.05-0.2 mg/day [20].

The most common issue with treating adrenocortical insufficiency is overtreatment. Excess GC replacement affects the quality of life and causes multiple morbidities and increased mortality [20]. Patients with long-term supraphysiological GC replacement have been found to be at risk for a higher incidence of infections, especially those of the upper airways and gastrointestinal tract, and show an increased rate of hospitalization [20]. None of the current GC preparations can adequately mimic physiology [18]. Currently, there are a few pressed pills of hydrocortisone that contain the correct pediatric dosages. This causes patients to have to split pills in half or crush the pressed pills, which leads to increased room for dosage error. Another barrier to correct dosage and treatment with GC replacement is that there are no biochemical parameters that can be used reliably for monitoring under- or overtreatment [18]. In current clinical practice, clinical judgment is used to assess the adequacy of treatment [18]. Patients should be assessed for adverse effects, including weight gain, glucose intolerance, moon face, double chin, thin skin, decreased bone mineral density, or osteoporotic fractures [18].

Another consideration is the treatment of adrenal crises. Adrenal crisis is an acute, life-threatening condition with a mortality rate of 0.5/100 [21]. Identification and treatment must be made by a clinician in a prompt and timely manner to prevent adverse outcomes. The mainstay of treatment for adrenal crises revolves around GC and fluid replacement, with GC’s choice of hydrocortisone given by continuous infusion instead of intermittent boluses [21]. In adults, 100 mg of hydrocortisone should be given IV/IM initially. Each additional 200 mg IV or 50 mg IM is divided into doses every six to eight hours for the next 24 hours [21]. Fluid resuscitation includes 1 liter of normal saline or 5% dextrose in 1 liter of normal saline in the case of hypoglycemia, followed by maintenance fluids. In children, the hydrocortisone dose is calculated as 50-100 mg per meter square followed by 50-100 mg per meter square over the next 24 hours given IM/IV (divided into doses given every six hours) or as a continuous infusion. Fluid recusation includes a normal saline bolus at a dose of 20 ml/kg of body weight, with repeated doses at up to 60 ml/kg during the first hour. Dextrose is added at a dose of 0.5-1 g per kilogram in the case of hypoglycemia [21].

Related to the adrenal cortex’s circadian rhythm being difficult to precisely replicate via GC replacement, there are several new treatments on the horizon [22]. Androgen/estrogen antagonists and synthesis inhibitors can be used with GC replacement in women to combat acne and hirsutism associated with CAH. Growth-promoting drugs have been studied as a treatment for reduced heights in patients with AI. However, normal height may be achieved with adequate GC replacement, making growth hormone replacement not a standard of care currently. Another possible treatment is an adrenalectomy. An adrenalectomy reduces virilization in females and permits decreased GC doses, lowering the adverse effects of GCs but raising the risk of adrenal crisis [22].

Alkindi Sprinkle

Alkindi Sprinkle is a pediatric-specific hydrocortisone oral granule that manages cortisol levels to help facilitate accurate, individualized dosing in infants, children, and adolescents (neonate to <17 years old) with AI, including CAH [23]. Alkindi was developed to help prevent inaccurate dosing in pediatric patients and stop adverse outcomes associated with incorrect doses that extend beyond childhood [23]. Alkindi Sprinkles are hydrocortisone granules that come in an openable cap, allowing precise dosages for pediatric patients [24]. Alkindi is available in flexible hydrocortisone dosages of 0.5 mg, 1 mg, 2 mg, and 5 mg, with its smaller dosage making it more appropriate for pediatric patients. This also allows for appropriate dose titration as the child grows. The maximum diameter of the granules is 0.8 mm, significantly smaller than the FDA’s guidance to industry on sprinkle formulation limits of 2.5 mm [24]. The small granule size decreases the risk of choking and allows easy swallowing of the granules by neonates [24]. Each granule has an inert cellulose capsule core, is sprayed with hydrocortisone, and is sealed with a taste masking coat that makes the granules less bitter. They are then contained within a size 00el transparent capsule that is meant to be opened to pour the granules out [24]. The clear capsule opens to allow for all granules in the capsule to be taken directly into the mouth or sprinkled onto soft food or yogurt [24].

To administer Alkindi, parents can gently squeeze the bottom of the capsule and twist off the top of the capsule [23]. Then, the HC granules can be poured directly onto the child’s tongue or poured onto a spoon of cold or room-temperature soft foods [23]. Immediately following administration, the patient should ingest fluids like milk, water, formula, or breast milk to ensure that all granules have been swallowed [23]. Alkindi should be swallowed and given within five minutes before the outer cover is dissolved to avoid any bitter tastes [23]. It is also not recommended for Alkindi to be added to granules, as this can also dissolve the outer taste masking cover [23].

Hydrocortisone is identical to human innate cortisol. After ingestion, the hydrocortisone is released from the granules and absorbed into the bloodstream. Once absorbed, hydrocortisone binds to GC receptors, migrates to the nucleus, and up- or downregulates gene expression. Cortisol is highly protein bound, with a preference for cortisol-binding globulin and a smaller amount of albumin binding. This leads to nonlinear pharmacokinetics as higher doses of hydrocortisone are more rapidly cleared due to saturation of the protein binding [24]. By providing a replacement for the deficient cortisol levels, Alkindi helps restore the physiological effects of cortisol in pediatric patients with AI. As with all GC replacement, the dosing of Alkindi should be carefully tailored to each patient based on their individual needs; close monitoring is necessary to ensure optimal hormone replacement therapy and prevent over- or underreplacement of cortisol. Toward this end, physiologically based pharmacokinetic models, as well as one predictive in vitro approach, have been developed to predict formulation pharmacokinetics and guide the dosing of formulations [25,26].

Hydrocortisone granules in capsules for opening are currently the only FDA-approved treatment for AI in the pediatric population. Our current understanding of the safety of steroid replacement stems from their use in high doses as anti-inflammatory agents. Using them in low doses makes the dose-dependent adverse events less relevant when using steroids for adrenal replacement with the goal of reproducing the natural physiology. A study published in 2021 showed that during Alkindi Sprinkle clinical trials, there were no serious adverse events reported, and all adverse events reported were mild or moderate [24]. The most commonly reported adverse outcomes of Alkindi Sprinkle were diarrhea, vomiting, and rash. The same study concluded that the Alkindi Sprinkle formulation of hydrocortisone was well tolerated without the occurrence of an adrenal crisis in clinical trials and was favored over current therapy by parents and children [24]. Contraindications for hydrocortisone granules include their use directly down a nasogastric (NG) tube due to the risk of blocking fine bore NG tubes [24]. Hypersensitivity to any component of the formula is a contraindication. Additional contraindications of corticosteroids, in general, include concurrent administration of live or live-attenuated vaccines (when using immunosuppressive dosages), systemic fungal infection, osteoporosis, uncontrolled hyperglycemia, diabetes mellitus, glaucoma, joint infection, uncontrolled hypertension, herpes simplex keratitis, and varicella infection [27].

Alkindi Sprinkle for the Treatment of AI

Alkindi Sprinkles are hydrocortisone granules used as hormone replacement in AI, specifically targeted toward children with conditions such as CAH who require replacement of deficient cortisol in various doses smaller than the adult 10-mg dose tablets. To administer this to children, a capsule filled with the granules is opened and emptied either onto soft foods (not mixed) or directly onto the child’s tongue and swallowed to be absorbed enterically to replace the deficient hormone (cortisol) in the body [9,10]. Oral hydrocortisone has been a well-established and effective treatment for AI in adults; however, pediatric treatment presents substantial obstacles. Prior to the granule formulation of hydrocortisone, pediatric dosing required crushing large adult dose tablets or compounding by a pharmacist - methods that both result in inaccurate dosing, which increases the risk of adverse side effects on growth, pubertal development, blood pressure, body mass index, cardiovascular disease, and bone density [6]. In a 2017 study, the majority of pediatric physicians rely on compounded adult medication due to the lack of licensed, dose-appropriate formulations for children with AI [7].

Because Alkindi is bioequivalent to its tablet-form predecessors, the physiologic profile of either formulation is essentially identical. A two-year prospective study of 17 children under the age of six with AI treated with hydrocortisone granules recorded no adrenal crises during this period [28]. A 2018 study of cortisol levels in three cohorts of children under six years of age (n = 24) with AI treated by Alkindi Sprinkle showed cortisol levels similar to those of physiological levels in healthy children at 60 minutes after administration [29]. This evidence strongly supports the drug’s efficacy. The primary distinguishing factor between the formulations is the accuracy of dosing via encapsulated granules filled by a pharmaceutical manufacturer to an exact quality- and quantity-assured dose versus the high risk of dose alteration through post-production interference via crushing or compounding [11,30].

In contrast, according to a 2017 study of compounded samples randomly collected from volunteers in Germany, up to 25% of hydrocortisone capsules compounded by pharmacies failed to meet the acceptance criteria of the European Pharmacopoeia for uniformity in dose/drug content [7]. A 2018 study measuring the contents of 10 mg of hydrocortisone tablets quartered by different operators using a pill splitter demonstrated that more than half of all the quartered 10-mg tablets resulted in hydrocortisone content outside of the +/-10% acceptable US Pharmacopeia variation [31]. Even giving the same labeled dose and original formulation resulted in “considerable” variations in cortisol exposure, according to one 2018 study [32]. These seemingly minor differences in doses can still have an enormous impact on the health of the children depending on exogenous hydrocortisone. A study of 51 young children being treated for AI via nongranule formulations (primarily quartered nondiluted 10-mg tablets) showed an average number of adrenal crisis episodes ranging from 4.9 to 7.3 per patient per year, the higher end being associated with younger children under four years of age; despite this, the two children, both of whom were under four years of age, being treated with the more accurate micronized weighted formulation obtained in a compounding pharmacy from 10-mg hydrocortisone tablets were reported to have no episodes of suspected adrenal crisis [11].

Even outside of the targeted application of the drug, hydrocortisone is associated with many adverse side effects, especially when administered in doses that are higher than desired. A cross-sectional study of 42 patients with PAI treated with standard hydrocortisone therapy showed impairment in multiple different mechanisms of the patient’s immune systems, including natural killer (NK) cell cytotoxicity, NK cell surface receptors, and ratios of classical vs. nonclassical monocytes [20,33]. GC levels higher than normal physiologic levels may also be associated with increased cardiovascular morbidity [5]. Even mental health, quality of sleep, and quality of life may be negatively impacted via the dysregulation of the cortisol-mediated circadian profile [5]. In support of the benefits of accurate dosing, a prospective study of children from zero to eight years old with CAH and AI treated with hydrocortisone granules in 2020 demonstrated that the more accurate dosing achieved through the granule formulation, along with monitoring from birth, resulted in “doses at the lower end of the recommended dose range and normal growth, without occurrence of adrenal crises” [28]. Accurate dosing is not the only benefit to Alkindi. Because the multiparticulate granule formulation passes through a 0.8-mm sieve during production, the maximum diameter of any granule remains well below the FDA sprinkle formulation limit of 2.5 mm, which allows even neonates to swallow the granules [6]. Additionally, the formulation’s taste masking may improve palatability and acceptance by young children, making administration easier and more convenient for their parents and contributing to increased adherence and compliance [29]. The drug can also be sprinkled on soft food for ease of administration.

Although there are many benefits to hydrocortisone granules, certain drawbacks also exist. A comparison of the different formulations’ costs revealed that the granules may be significantly more costly than compounded medications, which require an additional compounding fee on top of the cost of the medication itself due to the development and manufacturing processes involved in creating specialized formulations; even access to licensed pediatric granule formulations varies across different regions and may not be available to all patients who require them [6]. Also, as the granules cannot be dissolved, they may prove difficult to administer to infants who may dislike the texture of the drug, despite its taste masking [20]. Overall, available evidence strongly supports the efficacy and safety of Alkindi. Multiple studies have demonstrated the drug exhibited good absorption, palatability, and positive safety profiles and achieved cortisol levels similar to those in healthy children; furthermore, Alkindi Sprinkle is well tolerated, easy to administer, and can provide accurate doses for different age groups, although it may currently be more expensive than tablet formulations [6,29,21]. Table 1 summarizes the key features and outcomes of some of the significant studies referenced in this review.

**Table 1:** Key features and outcomes of certain studies surrounding the use of hydrocortisone in the treatment of pediatric AI AI, adrenal insufficiency

Study	Groups studied	Results	Conclusions
Whitaker et al. [21]	European healthcare providers and patients	Alkindi Sprinkle was well tolerated, bioequivalent, and dose-proportional with respect to cortisol exposure	Supports the use of Alkindi Sprinkle as an appropriate and effective treatment option for pediatric AI
Neumann et al. [28]	Seventeen children with AI (ages zero to eight)	No adrenal crises were observed nor accelerated or reduced growth in any children; 80%	Alkindi Sprinkle showed improved quality of life and overall treatment satisfaction, providing accurate dosing and addressing the limitations of compounded hydrocortisone tablets
Neumann et al. [29]	Twenty-four children with AI	A total of 95.5% of caretakers preferred Alkindi Sprinkle to their child’s current medication; 6/7 children rate palatability as “very good” or “neutral”	Alkindi Sprinkle was well tolerated, easy to administer, and showed good absorption with cortisol levels similar to healthy children
Neumann et al. [7]	Capsule samples from volunteering parents of treated German children	A total of 25% of tested batches failed quality standards due to net mass, drug content, or failure to contain labeled drug	Compounded medication can cause variation in steroid doses, risking poor disease control and adrenal crises, highlighting the need for licensed pediatric formulations approved by regulatory authorities
Madathilethu et al. [31]	Operators splitting hydrocortisone tablets	Over half of all quartered tablets resulted in dose variations outside the ±10% US requirement	Quartering 10-mg hydrocortisone tablets produced unacceptable dose variations, while mini-tablets provided more accurate doses for pediatric patients

In summary, adequate therapeutic treatment of pediatric patients with AI is difficult because of the lack of dose-appropriate hydrocortisone formulations. In this regard, many pediatric AI patients suffer from over- or undertreatment, resulting in adverse effects on growth, development, and metabolic health. However, recent advancements have led to the development of Alkindi Sprinkle. Alkindi Sprinkles are hydrocortisone granules used as hormone replacement in cases of AI, specifically targeted toward children with CAH. These granules come in an openable cap, allowing for precise dosage and appropriate dose titration as the child grows. Alkindi Sprinkle offers numerous advantages for the treatment of pediatric AI. Some of these advantages include accurate dosing, taste masking, ease of administration, and even reducing the likelihood of an adrenal crisis. Clinical studies have described the efficacy and safety of Alkindi Sprinkle in providing adequate hydrocortisone replacement and reducing the risk of adrenal crisis. These studies showed that Alkindi is well tolerated, easy to administer, and replenishes cortisol levels similar to those of healthy children. By providing an exact dose, Alkindi Sprinkle ensures pediatric AI patients receive appropriate treatment and improved patient outcomes.

## Conclusions

Overall, the development of Alkindi Sprinkle has addressed the challenge of accurate hydrocortisone dosing in pediatric AI patients. It provides a reliable treatment option that ensures adequate dosing in a way preferred for pediatric patients. The sprinkle formulation allows for ease of administration, and its taste masking properties allow for it to be well tolerated. However, further research and clinical studies are necessary to fully assess Alkindi Sprinkle’s long-term safety and efficacy. Alkindi Sprinkle has the potential to become the drug of choice in the treatment of pediatric patients with adrenocortical insufficiency, improving their health outcomes and overall quality of life.
